# Microsurgical Anatomy of the Labyrinthine Artery: A Cadaveric Microdissection Study of Number, Origin and Course at the Cerebellopontine Angle

**DOI:** 10.3390/medicina62061073

**Published:** 2026-06-01

**Authors:** Ionuț Isaia Jeican, Costel Vasile Siserman, Corneliu Toader, Veronica Elena Trombitaș

**Affiliations:** 1 National Institute for Research and Development of Isotopic and Molecular Technologies, 400293 Cluj-Napoca, Romania; 2 “Salvosan” Polyclinic, 400012 Cluj-Napoca, Romania; 3 Department of Legal Medicine, Iuliu Hațieganu University of Medicine and Pharmacy, 400006 Cluj-Napoca, Romania; 4 Institute of Legal Medicine, 400006 Cluj-Napoca, Romania; 5 Department of Neurosurgery, Carol Davila University of Medicine and Pharmacy, 050474 Bucharest, Romania; 6 Clinic of Neurosurgery, National Institute of Neurology and Neurovascular Diseases, 077160 Bucharest, Romania; 7 Department of Head and Neck Surgery and Otorhinolaryngology, Iuliu Hațieganu University of Medicine and Pharmacy, 400015 Cluj-Napoca, Romania; 8 Department of ENT, University Clinical Hospital of Railway Company, 400015 Cluj-Napoca, Romania

**Keywords:** labyrinthine artery, cerebellopontine angle, microdissection

## Abstract

*Background and Objectives*: The labyrinthine artery (LA) is a small but surgically important vessel encountered during procedures involving the cerebellopontine angle (CPA). Variations in its number, origin, and relationship to the facial–vestibulocochlear nerve complex may increase the risk of vascular injury during CPA surgery. The aim of this cadaveric microdissection study was to evaluate the number, origin, and course of the LA within the CPA and to characterize its relationship to the anterior inferior cerebellar artery (AICA) and the facial–vestibulocochlear nerve complex. *Materials and Methods*: Microsurgical dissections were performed bilaterally in 45 formalin-fixed adult cadavers (90 CPAs) using an operating microscope and vascular injection. The number, origin, and course of the LA were analyzed together with its relationship to the AICA and the facial–vestibulocochlear nerve complex. *Results*: The LA was identified in all specimens. A single LA was observed in 57.8% of CPAs, whereas multiple LAs were identified in 42.2%. The artery most commonly originated from the AICA (65.6%), followed by a common trunk shared with the AICA (21.1%) and direct origin from the basilar artery (13.3%). In specimens with bifurcated AICAs forming double loops, multiple LAs frequently arose from both loops. Considerable variability was observed in the course of the LA relative to the facial–vestibulocochlear nerve complex, including superior, inferior, and interposed courses. Bilateral asymmetry was identified in 15.6% of cadavers. *Conclusions*: The LA demonstrates substantial anatomical variability within the CPA regarding its number, origin, and neurovascular relationships. Although the artery most commonly arises from the apex or medial aspect of the AICA loop, its subsequent course may vary considerably. Careful microsurgical inspection remains essential during CPA surgery, as the configuration of the AICA alone is insufficient to reliably predict the distal trajectory of the LA.

## 1. Introduction

The labyrinthine artery (LA) is a small vessel of considerable anatomical and surgical significance encountered during microsurgical procedures involving the cerebellopontine angle (CPA) [1,2]. This narrow neurovascular corridor contains the facial (VII) and vestibulocochlear (VIII) nerves together with complex arterial relationships involving the anterior inferior cerebellar artery (AICA) and the LA. Vascular injury within this region may result in significant functional morbidity [3].

The LA represents the principal arterial supply to the cochlea and vestibular labyrinth and provides vascular supply to the facial and vestibulocochlear nerves [4,5,6]. In most individuals, the artery originates from the AICA, although variations in origin, branching pattern, and anatomical relationships with adjacent cranial nerves have been described [1,7,8,9,10,11,12].

Due to its intimate relationship with the VII–VIII nerve complex and the internal acoustic meatus, the LA may be particularly vulnerable during CPA surgery, especially in vestibular schwannoma procedures where the regional neurovascular anatomy is frequently distorted [13,14,15,16,17,18]. Injury or vasospasm involving the LA may result in hearing loss, vertigo, tinnitus, or other vestibulocochlear deficits [19,20,21,22].

Although several anatomical studies have described the LA and the AICA within the CPA, the relationship between AICA loop morphology, LA origin, and the subsequent course of the artery relative to the facial–vestibulocochlear nerve complex remains variably reported in the literature [1,8,11]. In particular, the extent to which the distal trajectory of the LA may be inferred from the configuration of the AICA loop remains insufficiently clarified.

The aim of the present cadaveric microdissection study was to evaluate the number, origin, and course of the LA within the CPA and to characterize its relationship to the AICA and the facial–vestibulocochlear nerve complex.

## 2. Materials and Methods

### 2.1. Specimens

This study was conducted on 45 adult human cadavers (corresponding to 90 CPAs), obtained through the Cadaver Use Service of the “Iuliu Hațieganu” University of Medicine and Pharmacy, Cluj-Napoca, Romania, and used in accordance with ethical and legal standards (Law 104/2003 on the handling of bodies and the removal of organs and tissues with a view to transplantation). The research protocol for this study was approved by the Ethics Committee of “Iuliu Hațieganu” University of Medicine and Pharmacy, Cluj-Napoca, Romania under No. 111/2023.

All cadavers were bilaterally injected with 10% formalin via the common carotid arteries at least 5 days prior to dissection. Following craniotomy and removal of the dura mater, a transverse section was performed at the level of the pons to remove the supratentorial brain structures. The basilar artery was then isolated, and its contents were aspirated using a cannula connected to a vacuum pump. Subsequently, the basilar artery was injected retrogradely with approximately 1 mL of colored aqueous solution. The basilar artery was clamped proximally using a Pean forceps, and a period of 30 min was allowed for the colored solution to disperse throughout the arterial system.

Specimens presenting evidence of prior cranial surgery, trauma, or pathological lesions involving the posterior fossa were excluded.

### 2.2. Dissection Technique

Microsurgical dissections were performed using an operating microscope (Carl Zeiss Inc., Göttingen, Germany) at magnifications ranging from ×5 to ×32. Following standard cranial opening procedures and dural removal, the cerebellopontine angle was exposed bilaterally. Cerebrospinal fluid release was used to facilitate gentle cerebellar relaxation without fixed retraction.

The arachnoid membranes of the CPA were carefully dissected to expose the facial (VII) and vestibulocochlear (VIII) nerves, the AICA, and the LA. Vascular structures were identified under microscopic magnification and followed toward the internal acoustic meatus whenever anatomical preservation allowed adequate visualization.

### 2.3. Anatomical Analysis

For each specimen, the following parameters were systematically documented:

• Presence and number of the LA;
• Site of origin and parent vessel;
• Relationship of the LA to the AICA loop;
• Course of the LA relative to the facial–vestibulocochlear nerve complex.

The LA was identified using a combination of anatomical criteria, including its site of origin, trajectory toward the internal acoustic meatus, relationship to the VII–VIII nerve complex, and continuity and caliber under microscopic magnification. Small perforating vessels, vasa nervorum, and branches morphologically consistent with the subarcuate artery were excluded from classification as LAs.

Anatomical findings and vessel classifications were reviewed by two investigators. In cases of uncertain vessel identification or classification, a consensus evaluation was performed.

Neurovascular relationships were described using a superior–inferior orientation relative to the facial–vestibulocochlear nerve complex.

### 2.4. Documentation and Classification

High-resolution photographs were obtained during the cadaveric microsurgical dissections using a digital camera attached to the operating microscope. Representative specimens demonstrating the main anatomical configurations were selected for photographic documentation.

Anatomical configurations were classified according to AICA loop morphology, number of LAs, and the relationship of the LA to the facial–vestibulocochlear nerve complex.

### 2.5. Data Analysis

Descriptive statistics were used to summarize the anatomical findings. Frequencies, percentages, and 95% confidence intervals (95% CI) were calculated for categorical variables, including the number, origin, and course of the LA, as well as its relationship to the facial–vestibulocochlear nerve complex.

Given the descriptive anatomical design of the study and the heterogeneity of vascular configurations, no inferential statistical analysis was performed.

## 3. Results

### 3.1. Presence and Number of the LA

The LA was identified in all 90 CPAs examined (100%).

**Figure 1:**
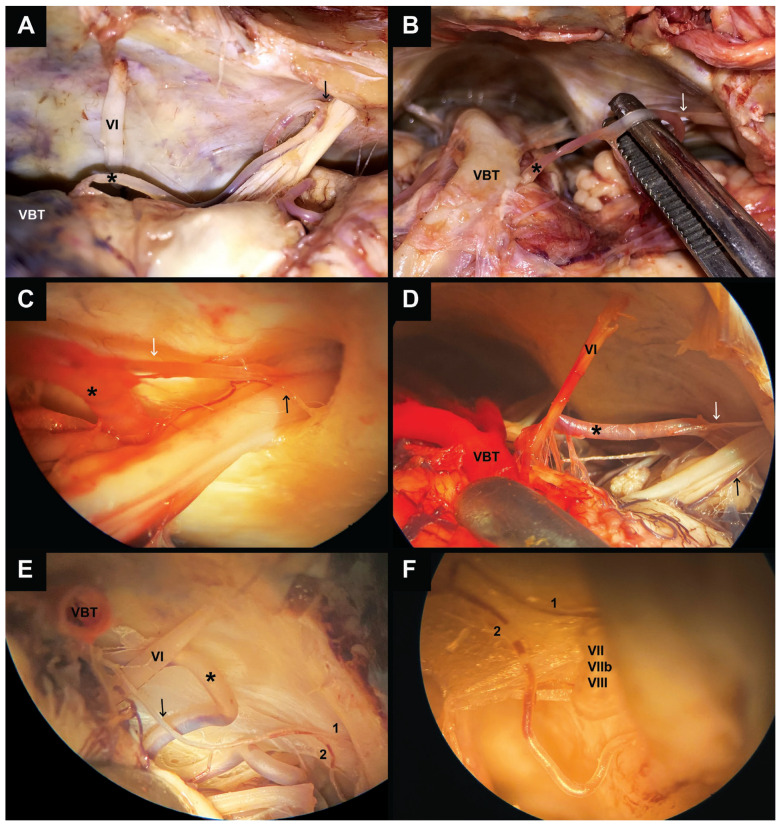
Origin of a single LA. VBT—Vertebrobasilar trunk, *—AICA, VI—abducens nerve, VII—facial nerve, VII_b_—intermediate nerve (nerve of Wrisberg), VIII—vestibulocochlear nerve. (**A**): The LA (arrow) originates from the apex of the AICA loop. The loop is positioned superior to the facial–vestibulocochlear nerve complex, whereas the LA courses inferior to the nerve complex. (**B**): The LA (arrow) originates from the apex of the AICA loop. Both the AICA loop and the LA are located superior to the facial–vestibulocochlear nerve complex. The nerve complex has been resected. (**C**): The LA (white arrow) originates from the apex of the AICA loop and courses between the facial and vestibulocochlear nerves. The loop is positioned inferior to the facial–vestibulocochlear nerve complex. LA gives rise to a collateral branch (black arrow) supplying the perimeatal meninges. (**D**): The LA (white arrow) originates medial to the apex of the AICA loop. The loop is positioned inferior to the facial–vestibulocochlear nerve complex, whereas the LA courses superior to the nerve complex. Labyrinthine vein (black arrow). (**E**): The LA (arrow) originates from the basilar trunk and crosses superior to the abducens nerve (VI). Multiple loops are observed along the course of the AICA. The LA bifurcates into two branches (1 and 2). The facial–vestibulocochlear nerve complex has been resected. (**F**) (the same CPA, detail): At the level of the internal acoustic meatus, the LA bifurcates into two branches (1 and 2).

A single LA was observed in 52 CPAs (57.8%; 95% CI: 47.5–67.5) (Figure 1).

**Figure 2:**
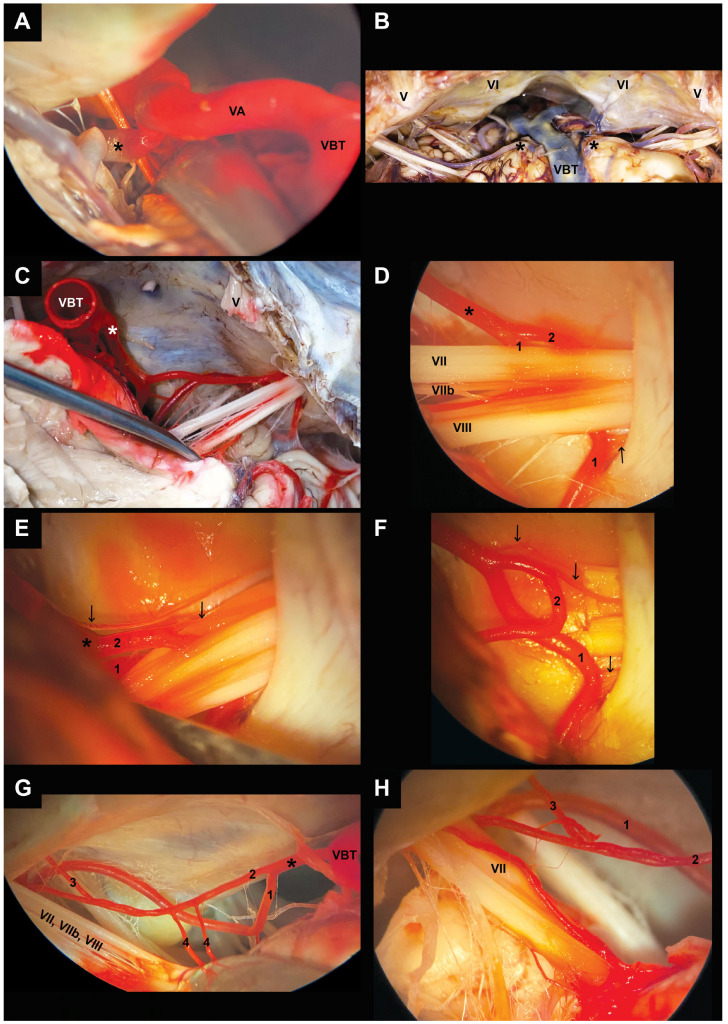

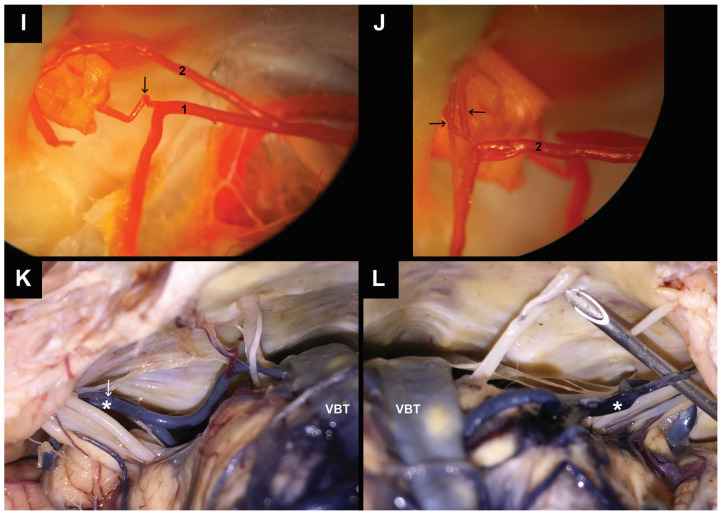
AICA configurations and associated LA anatomy within the CPA. VA—Vertebral artery, VBT—Vertebrobasilar trunk, *—AICA, V—trigeminal nerve, VI—abducens nerve, VII—facial nerve, VII_b_—intermediate nerve (nerve of Wrisberg), VIII—vestibulocochlear nerve. (**A**): Origin of the AICA from the vertebral artery. (**B**): Left–right asymmetry of AICA morphology. On the right side, the AICA loop is located superior to the facial–vestibulocochlear nerve complex, whereas on the left side it courses between the facial and vestibulocochlear nerves. (**C**): AICA loop extending into the internal acoustic meatus. (**D**): Bifurcation of the AICA forming two distinct loops. Loop no. 2 courses between the facial and vestibulocochlear nerves and is positioned superior to loop no. 1, which lies inferior to the nerve complex. The origin of a LA (arrow) is visible at the apex of loop no. 1. (**E**): (the same CPA): Prior to AICA bifurcation, a common trunk is identified, from which a LA (arrow) arises. A second LA (arrow) originates medial to the apex of loop no. 2. (**F**): (the same CPA): Origin of three LAs: two arising from the loops of the AICA and one originating from a common trunk shared with the AICA. The nerve complex has been resected. (**G**,**H**): Bifurcation of the AICA into two loops. Loop no. 2 courses between the facial and vestibulocochlear nerves and is positioned superior to loop no. 1, which lies inferior to the nerve complex. 3—Subarcuate artery; 4—Pontine branches. (**I**): (the same CPA, detail): A LA (arrow) arising from the apex of loop no. 1. The nerve complex has been resected. (**J**): (the same CPA, detail): Two LAs (arrows) arising from the apex of loop no. 2. The nerve complex has been resected. (**K**,**L**): (the same cadaver): Bilateral bifurcation of the AICA forming two loops on each side: a superior loop coursing between the facial and vestibulocochlear nerves and an inferior loop located below the nerve complex. Prior to bifurcation, a common trunk is present on both sides, from which a LA arises. On the right side, the LA is positioned superior to the nerve complex, whereas on the left side the LA (arrow) courses inferiorly.

**Figure 3:**
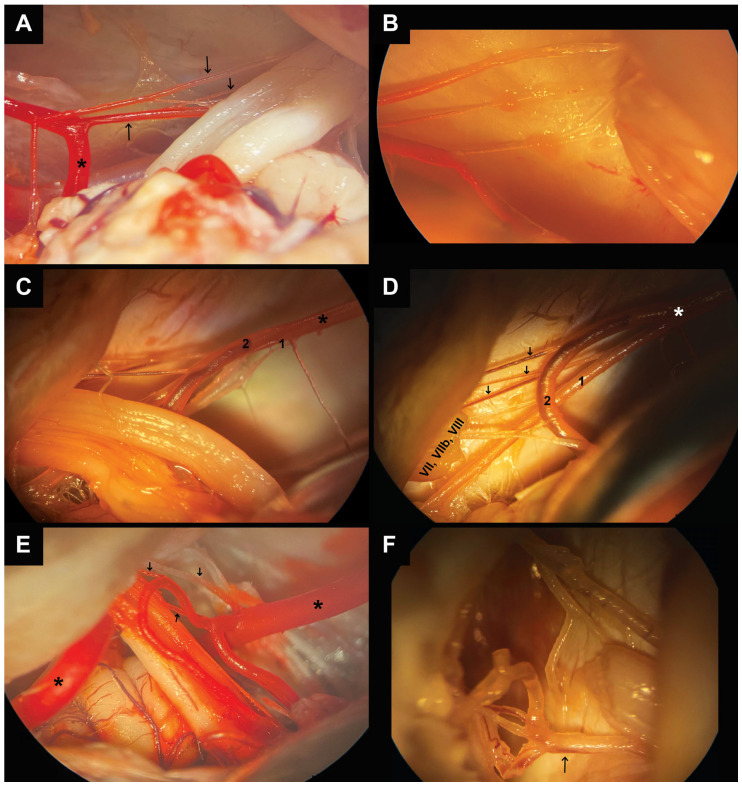
Multiple LAs: origin and course within the CPA. *—AICA, VII—facial nerve, VII_b_—intermediate nerve (nerve of Wrisberg), VIII—vestibulocochlear nerve. (**A**): Multiple LAs arising from the apex of the AICA loop and medial to it (arrows). (**B**) (the same CPA, detail): Branching of the LAs at the level of the internal acoustic meatus. The facial–vestibulocochlear nerve complex has been resected. (**C**,**D**): The AICA bifurcates and forms two branches (1 and 2) with an inferior course relative to the facial–vestibulocochlear nerve complex. (**D**): Arrows indicate the LAs, whose course can be observed after resection of the nerve complex. (**E**): The AICA forms one loop superior and one loop inferior to the facial–vestibulocochlear nerve complex. The origins of multiple LAs are indicated by arrows. (**F**): Complex anastomotic configuration of the LA (arrow).

Multiple LAs were identified in 38 CPAs (42.2%; 95% CI: 32.5–52.5). Figure 2E,F show the same CPA with three LAs: two arising from the loops of the AICA and one originating from a common trunk shared with the AICA. Figure 2I,J show the same CPA with three LAs: one arising from one AICA loop and two originating from the other AICA loop. Figure 3A–F show multiple LAs with variable origin and course across four CPAs from different cadavers.

Complete absence of the LA was not observed in any specimen.

### 3.2. Origin of the LA

The LA most commonly originated from the AICA (59/90 CPAs, 65.6%; 95% CI: 55.3–74.6). The origin was typically located at the apex of the AICA loop (Figure 1A–C and Figure 2F,I each illustrating different CPAs) or medial to the loop apex (Figure 1D and Figure 3D,E each illustrating different CPAs).

A direct origin from the basilar artery was observed in 12 CPAs (13.3%; 95% CI: 7.8–21.9) (Figure 1E). In 19 CPAs (21.1%; 95% CI: 14.0–30.7), the LA arose from a common trunk shared with the AICA (Figure 2E and Figure 3E). Figure 2K,L show that a common trunk is present on both sides for the same cadaver.

No origin from the posterior inferior cerebellar artery was identified. Mixed origins involving both the AICA and basilar artery were not observed.

### 3.3. Course of the LA Within the CPA and Neurovascular Relationships

The course of the LA within the CPA varied in relation to both the facial–vestibulocochlear nerve complex and the AICA (Figure 1, Figure 2 and Figure 3).

In specimens with a single LA (52/90 CPAs, 57.8%), the artery most frequently followed a course on the same side of the facial–vestibulocochlear nerve complex as the AICA loop (40/52 CPAs, 77%). In these specimens, the LA was located superior to the nerve complex (Figure 1B), passed between the facial and vestibulocochlear nerves, or coursed inferior to the nerve complex (Figure 2K).

In the remaining 12/52 CPAs (23%), the course of the LA differed from the position of the AICA loop relative to the nerve complex. Figure 1A demonstrates a superiorly positioned AICA loop with an inferior course of the LA relative to the nerve complex. In Figure 1C, the AICA loop is positioned inferior to the facial–vestibulocochlear nerve complex, and the LA courses between the facial and vestibulocochlear nerves. In Figure 1D, the AICA loop is positioned inferior to the nerve complex, whereas the LA courses superiorly.

In specimens with multiple LAs (38/90 CPAs, 42.2%) (Figure 3), 15/38 CPAs (39.5%) showed a common trunk shared with the AICA (Figure 2E and Figure 3E), whereas 23/38 CPAs (60.5%) were associated with a bifurcated AICA forming double loops (Figure 2F). Figure 3C,D show the same CPA. In these specimens, LAs arose from both loops, with at least one artery originating from each loop. The total number of LAs ranged from two to three per CPA.

The LA exhibited close neurovascular relationships with both the facial and vestibulocochlear nerves, particularly near the internal acoustic meatus. In specimens with multiple LAs, at least one branch crossed either the facial or vestibulocochlear nerve.

### 3.4. Relationship Between the LA and the AICA Loop Configuration

In most specimens, the LA originated either from the apex of the AICA loop or medial to it, irrespective of the subsequent course of the artery relative to the facial–vestibulocochlear nerve complex.

In specimens with bifurcated AICAs forming double loops, multiple LAs arose from both loops (Figure 2D–J and Figure 3C–E). In these cases, the LAs demonstrated variable courses within the CPA relative to the facial–vestibulocochlear nerve complex.

No consistent differences were observed between left- and right-sided CPAs regarding the origin, number, or overall course of the LA on descriptive analysis. However, intraindividual left–right asymmetry was identified in 7/45 cadavers (15.6%), in which the configuration of the LA differed between the two sides (Figure 2B,K,L). These differences most commonly involved variations in the origin and number of the LAs, their relationship to the facial–vestibulocochlear nerve complex, and the morphology of the AICA loops.

## 4. Discussions

The number of LAs (Figure 1, Figure 2 and Figure 3) demonstrates considerable variability across the anatomical literature, with reported configurations ranging from single to duplicated, triplicated, or even quadruplicated vessels. While several anatomical series describe a single LA as the most frequent finding, other studies identify duplication as the predominant variant, likely reflecting differences in dissection techniques, sample size, and methodological approaches [1,2,4,7].

Our results are generally consistent with the most recent systematic review of the literature (33 studies, *n* = 3778 LAs), which reported a single LA in 51% of cases and an origin from the AICA in 75.4% of cases [1]. In the present study, a single LA was identified in 57.8% of CPAs, whereas an origin from the AICA was observed in 65.6% of specimens. However, substantial variability between anatomical series remains evident in the literature.

The anatomical variability of the AICA itself likely contributes to differences in the origin and course of the LA. The AICA demonstrates highly variable loop configurations within the CPA, including duplicated or complex looping patterns and intrameatal extensions [23,24,25,26]. In the present study, bifurcated AICAs forming double loops were consistently associated with multiple LAs arising from both loops. These observations suggest that evaluation of LA anatomy should also be performed in the context of the parent AICA morphology.

From a microsurgical perspective, the presence of multiple LAs may complicate the identification of vascular structures within the CPA, particularly during vestibular schwannoma surgery or procedures involving extensive arachnoid dissection. In such settings, distorted neurovascular anatomy may increase the risk of inadvertent arterial injury, especially when vascular mobilization or tumor dissection is performed near the internal acoustic meatus.

**Figure 4:**
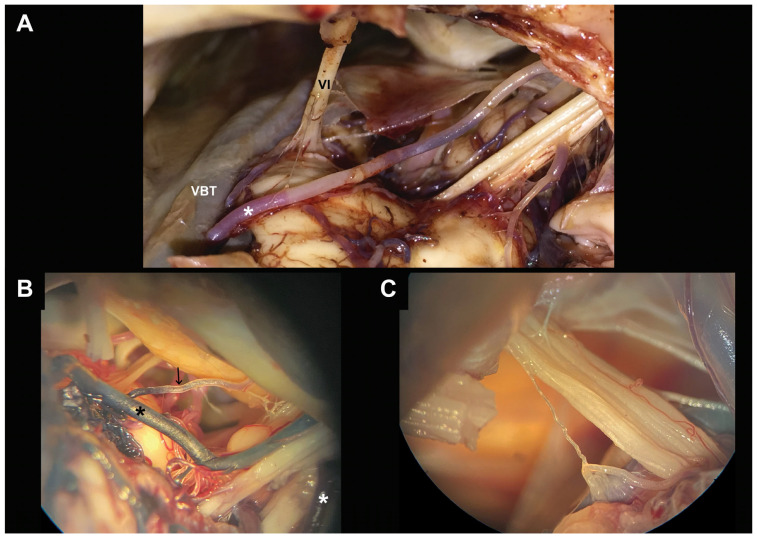
Particular anatomical features and potential confounding factors. VBT—Vertebrobasilar trunk, *—AICA, VI—abducens nerve. (**A**): The AICA forms a complex loop coursing between the facial and vestibulocochlear nerves. (**B**): The subarcuate artery (arrow). (**C**): Vasa nervorum in the vicinity of the internal acoustic meatus.

AICA loops (Figure 2 and Figure 4A) have also been implicated in possible neurovascular compression syndromes involving the vestibulocochlear nerve complex [27,28]. Consequently, attempts at vascular mobilization or microvascular decompression should be performed with caution, as injury or vasospasm involving the LA may result in irreversible auditory or vestibular deficits.

Although the LA most commonly originated from the apex or medial aspect of the AICA loop, its subsequent course relative to the facial–vestibulocochlear nerve complex demonstrated considerable variability. Similar AICA configurations were associated with different arterial trajectories, suggesting that the morphology of the parent vessel alone is insufficient to reliably predict the distal course of the LA within the CPA. These findings support the importance of careful intraoperative inspection of the neurovascular anatomy rather than reliance on presumed arterial trajectories.

Several limitations of the present study warrant consideration. Although cadaveric microdissection provides direct visualization and detailed anatomical documentation of the LA, it cannot fully reproduce in vivo conditions. Vessel diameter, pulsatility, and dynamic neurovascular relationships are inherently altered after death. Formalin fixation and postmortem changes may also reduce the detectability of very small-caliber branches, potentially leading to underrepresentation of fine secondary LAs.

Despite the use of vascular injection techniques, arterial visualization may have been influenced by technical factors, including variability in injection pressure, incomplete filling in specimens with advanced atherosclerotic changes, and differences in vessel patency. In addition, identification of the LA may be partially operator-dependent, particularly when differentiating it from the subarcuate artery (Figure 4B), vasa nervorum (Figure 4C), or small AICA branches.

Finally, although the sample size is substantial for a cadaveric anatomical study, the present findings remain descriptive in nature and cannot establish functional or hemodynamic correlations. The results should therefore be interpreted as detailed anatomical observations intended to improve microsurgical understanding and intraoperative awareness within the CPA.

## 5. Conclusions

The labyrinthine artery demonstrates considerable anatomical variability within the cerebellopontine angle regarding its number, origin, and relationship to the facial–vestibulocochlear nerve complex. Although the artery most commonly arises from the apex or medial aspect of the AICA loop, its subsequent course within the CPA may vary substantially.

The presence of multiple LAs, bifurcated AICA loops, and variable neurovascular relationships highlights the importance of careful microsurgical inspection during CPA procedures. Identification of the AICA loop alone should not be considered sufficient for predicting the distal trajectory of the LA relative to cranial nerves VII and VIII.

These findings emphasize that the microsurgical anatomy of the LA cannot be reliably inferred from simplified anatomical models or presumed neurovascular relationships. A precise, case-specific intraoperative assessment remains essential for safe CPA surgery, particularly in procedures aimed at hearing preservation, where inadvertent injury to this small but critical vessel may result in irreversible auditory or vestibular deficits.

## Data Availability

The data presented in this study are available on request from the corresponding author (the data are not publicly available due to privacy and ethical restrictions).

## Abbreviations

The following abbreviations are used in this manuscript:

LA	Labyrinthine artery
CPA	Cerebellopontine angle
AICA	Anterior inferior cerebellar artery
VBT	Vertebrobasilar trunk
VA	Vertebral artery
